# Implementation of modified team-based learning within a problem based learning medical curriculum: a focus group study

**DOI:** 10.1186/s12909-018-1172-8

**Published:** 2018-04-10

**Authors:** Annette Burgess, Chris Roberts, Tom Ayton, Craig Mellis

**Affiliations:** 1 Sydney Medical School – Education Office, The University of Sydney, Edward Ford Building A27, Sydney, NSW 2006 Australia; 2 Sydney Medical School – Central, Sydney Medical School, The University of Sydney, Sydney, NSW Australia

**Keywords:** Team-based learning, Problem based learning, Medical school

## Abstract

**Background:**

While Problem Based Learning (PBL) has long been established internationally, Team-based learning (TBL) is a relatively new pedagogy in medical curricula. Both PBL and TBL are designed to facilitate a learner-centred approach, where students, in interactive small groups, use peer-assisted learning to solve authentic, professionally relevant problems. Differences, however, exist between PBL and TBL in terms of preparation requirements, group numbers, learning strategies, and class structure. Although there are many similarities and some differences between PBL and TBL, both rely on constructivist learning theory to engage and motivate students in their learning. The aim of our study was to qualitatively explore students’ perceptions of having their usual PBL classes run in TBL format.

**Methods:**

In 2014, two iterations in a hybrid PBL curriculum were converted to TBL format, with two PBL groups of 10 students each, being combined to form one TBL class of 20, split into four groups of five students. At the completion of two TBL sessions, all students were invited to attend one of two focus groups, with 14 attending. Thematic analysis was used to code and categorise the data into themes, with constructivist theory used as a conceptual framework to identify recurrent themes.

**Results:**

Four key themes emerged; guided learning, problem solving, collaborative learning, and critical reflection. Although structured, students were attracted to the active and collaborative approach of TBL. They perceived the key advantages of TBL to include the smaller group size, the preparatory Readiness Assurance Testing process, facilitation by a clinician, an emphasis on basic science concepts, and immediate feedback. The competitiveness of TBL was seen as a spur to learning. These elements motivated students to prepare, promoted peer assisted teaching and learning, and focussed team discussion. An important advantage of PBL over TBL, was the opportunity for adequate clinical reasoning within the problem solving activity.

**Conclusion:**

Students found their learning experience in TBL and PBL qualitatively different. There were advantages and disadvantages to both. This suggests a hybrid approach utilising the strengths of both methods should be considered for wide scale implementation.

## Background

Introduced over half a century ago, problem-based learning (PBL) has long been accepted as a cornerstone in many medical education curricula worldwide. However, in re-designing curricula, medical educators need to balance producing graduates with contemporary attributes, where the learning environment is resource challenged, in both the higher education and health sectors. Team-based learning (TBL) has been proposed as a potential instructional method which can retain the educational strengths of PBL, and achieve these in a more efficient way. One of the attractions of TBL is that using fewer tutors solves many resource challenges, including rising student numbers and limited availability of clinicians to teach [1, 2]. What has not been fully explored, is the learning strategies that students use when taking part in TBL, and how these compare with the strategies students use in PBL. This study addresses the question of the student learning experience within a PBL curricula that was piloting the use of TBL. By understanding students’ learning experiences, future iterations of TBL may demonstrate improved outcomes [3].

PBL is a powerful learning approach offering many benefits including increased acquisition and retention of knowledge, stimulation of problem solving, enhancement of intrinsic subject interest, [4, 5] deep learning, enhanced communication, teamwork, presentation and critical appraisal skills [5, 6] independent learning, and enhanced clinical skills [7, 8]. However, concerns have been raised about achievements in basic science knowledge compared with traditional curricula [7, 8], because of persistent lower scores on basic science knowledge tests.

Team-based learning (TBL), is a relatively new pedagogy to medical education, but has gained popularity within the last 10 years [9]. Similar to PBL, TBL allows the integration of active learning strategies, predominantly [9] into the pre-clinical medical curriculum. Both PBL and TBL utilise collaborative learning methods to promote both critical thinking and team building skills – skills that are essential to medical students’ future work [10]. Both PBL and TBL provide learner-centred approaches to teaching that encourage students to work together to solve problems that are professionally relevant. Both approaches to learning ensure that learners use these problems to build on existing knowledge, and apply new knowledge [11].

TBL, however, is distinctive in both its design and highly structured format. In TBL one tutor facilitates a number of groups simultaneously, in one large room, making it suitable for large class teaching. In PBL, one tutor facilitates one group at a time, in small tutorial rooms. In PBL, no specific nor prescribed pre-reading is usually required. Students prepare as a group, and no formative assessment of learning prior to the tutorial takes place. In TBL, individual pre-reading is required, and to ensure the pre-reading has been completed, both an individual multiple choice test (Individual Readiness Assurance Test: IRAT) and a team pre-test (Team Readiness Assurance Test: TRAT), take place at the commencement of class [11].

Although there are many similarities and some differences between PBL and TBL, both rely on constructivist learning theory [12] to engage and motivate students in their learning. Both models (PBL and TBL) encourage active, participatory learning through problem solving, critical reflection, and guidance from a facilitator [12]. Constructivist learning theory posits that the acquisition of knowledge is structured by personal experiences, with new experiences adding and modifying one’s previous understanding [13]. Accordingly, in both PBL and TBL, students are provided with opportunities to challenge concepts and develop their understanding. Relevant problems and social interaction within small groups, and opportunities for reflection are provided [12].

While TBL is thought to have many similarities to PBL [14], to date, there has been little empirical evidence that students participating in the TBL model can engage with problems in a collaborative, and self-directed way, that enhances their critical thinking, and integrates biomedical and clinical concepts.

The aim of our study was to qualitatively explore our students’ experience of participating in the TBL class format, having previously been exposed to PBL. We use the conceptual framework of constructivist theory to guide our research and present our findings.

## Methods

### Study context

Sydney Medical School provides a four-year graduate entry program. In years 1 and 2, the curriculum is composed of organ-system based teaching blocks. At the time of the study Sydney Medical School used a two tutorial-based system in the first 2 years of the course for PBLs. This approach to PBL has been modified from the traditional PBL format. Working in collaboration with group members, students analyse a problem of practice, formulate hypothesis, and undertake self-directed learning to try to understand and explain all aspects of the PBL problem. The explanations are encouraged to be in the form of an underlying process, principle, or mechanism. The two 1.5 h tutorials are held on the same day, immediately following each other. The first tutorial being student-led, using the extensive IT materials to support the development of the case. The second tutorial is tutor led. The remainder of the students’ teaching and learning for each week was structured around the problem of the week, consisting of lectures, laboratory practical sessions, and hospital based clinical tutorials.

In our study, we piloted two consecutive iterations of TBL, in place of students’ usual PBL sessions towards the end of Year 1 in 2014, during an 8 week Cardiovascular Systems Block. The topics covered in PBL and TBL were:

Problem based learning (PBL): 1) Valvular heart disease; 2) Myocardial ischaemia; and 3) Heart failure.

Team-based learning (TBL): 1) Congenital heart disease, Down syndrome; and 2) Syncope and arrhythmia/hypertension.

### Participants

In total, there were 298 medical students enrolled in Year 1 at the time of the study (2014). Their PBL groups were permanent for the year. Twenty first year medical students participated in the study. Convenience sampling was used to select two established PBL groups of 10 students. One of the authors (CM), a paediatric respiratory physician, ran two tutorials in PBL format, and then two tutorials in TBL format, across four consecutive weeks.

### Structure of the TBL

While the learning outcomes remained the same, two iterations of PBL were converted to TBL format, and were designed to run for 1.5 h each. Teams of five were allocated by the researchers to ensure there was a diverse mix of students in each team. TBL clinical cases included: week 1: Infant with Down Syndrome and congenital heart disease, and Week 2: Young adult with sudden cardiac arrest from prolonged QT interval. We followed a modified TBL structure of:

1. Pre-reading: Prior to class, students were allocated compulsory readings to complete.
2. Readiness Assurance Test process (in total, 20 min duration):

At the beginning of each class, students’ individual knowledge of the pre-reading was assessed. We ran an Individual Readiness Assurance Test (IRAT) (10 min duration), followed by a Team Readiness Assurance Test (TRAT) (10 min duration). The test consisted of 10 Multiple-choice Questions (MCQs), each with one single best answers (SBA). Immediately on completion if the IRAT, the same MCQ test was then repeated by the students as a team (TRAT), with the intent of promoting discussion to establish team consensus.

3) Immediate feedback (20 min duration): The correct answers were then released, giving immediate feedback on team and individual responses. Thereafter, the facilitator offered clarification, particularly where teams had experienced difficulty, or disputes.
4) Problem solving activities (45 min duration): Students then worked in their teams on their problem-solving activity, utilising the knowledge from the pre-reading. Students had five clinical problems/tasks to work through in their teams. Examples of problems include: “Synthesise your thoughts into a summary statement about the patient, suitable for reporting to an Emergency Department Supervisor”. The final problem/task required student teams to draw a mechanistic flow chart on butcher’s paper using coloured pens.

It should be noted that peer evaluation was not part of our modified TBL format.

### Data collection and analysis

After completion of the two TBL sessions, all participating students were invited to immediately attend one of two focus group sessions (facilitated by AB and TA), with 14/20 (70%) participating. Data were transcribed verbatim. Thematic analysis was used to code and categorise the data into themes. Emergent themes in the dataset resonated closely with key constructs of constructivist theory. Subsequently constructivist theory was used as a conceptual framework to identify recurrent themes [15]. Authors AB, TA and CR coded a sample of the data, in order to identify recurrent themes and sub-themes. Once meaning had been negotiated and agreed between all the researchers, the first author applied the coding framework to all of the data [15].

Ethics approval was obtained from The University of Sydney Human Research Ethics Committee, project no. 2014/687.

## Results

Results are presented as the emergent prevalent themes, mapped to the conceptual framework of constructivist theory around guided learning, problem solving, collaborative learning and critical reflection.

### Guided learning

Students perceived that the knowledge and experience of PBL tutors varied greatly. As a result, they felt that their learning was highly dependent on which particular PBL group they had been allocated. Because of the emphasis within PBL on clinical reasoning, students who had had non-clinician facilitators expressed preference for clinicians as facilitators.

*“We had a physiology PhD student for our first block (of PBL). And we didn’t know because we were starting out as well, but it was a bit of a struggle. None of us knew what we were doing at all. And none of us knew anything about medicine … neither did they. I’d always prefer a clinician. … but there are a lot of PBLs run in a day, so it’s a manpower thing”.*

Consequently, students preferred the standardisation of the TBL approach, and points of clarification from the tutor were important to their learning, particularly around the depth and breadth of basic science relevant to a clinical problem. Guidance from a clinician facilitator also helped to focus the TBL sessions, with some students feeling that this was a more efficient way to meet their learning goals in relation to a specific problem scenario.

*“The good thing about the TBLs is that we can make sure that the information we get is correct….PBLs can sometimes go off on a massive tangent that isn’t really relevant to the case. I learnt more in TBL than PBL. It could be effective with a bit more refinement.”*

### Problem solving

Some students felt that within PBL, they weren’t able to develop the depth of knowledge in the basic science required to understand and work effectively on the clinical problem. Within TBL, students appreciated the structure whereby an appropriate emphasis was placed on basic science, in order to apply and link this knowledge to the problem solving activity.

*“In TBL because we went through the patho-physiology at the beginning with the questions, it made it much easier to link together. I think it went so much faster than our normal mechanism because we had end points that we had to link up”.*

For other students, the problem solving step within the TBL did not give the same opportunity for discussion on clinical reasoning compared to PBL.

*“I felt like there were some parts in the PBL that got a lot more focus that we didn’t find so much in the TBL. Like working the management side of things which we didn’t do a great deal on (in TBL).”*

### Collaborative learning

In TBL, the individual and team tests appeared to be an important influence in ensuring students came to class prepared relative to the rest of their team. Reasons given by the students included not wanting to let their team down, and feeling a sense of friendly competitiveness.

*“Even though it didn’t matter, us being all competitive people, you were motivated because you didn’t want to completely fluff the test. I think everyone did a bit of preparation”.*

The team test in TBL promoted competition between teams, and active discussion within teams.

*“I just took it (TBL) more seriously but I studied for it too. I actually took it more seriously the second week. I mean, last week, I’d say something and if somebody else would say something else, I’m like, okay, we’ll just go with that. But this week, I’m like, oh, wait, let’s think about that. We want to win”.*

This feature of competitiveness spurring learning in TBL is in direct opposition of PBL goals, which suggest that a lack of competitiveness is more likely to enhance collaborative learning [16, 17].

As in other settings, the size of the year cohort had outgrown the supply of PBL tutors [18]. The small size of the TBL groups (five students per team) compared to PBL groups (10 students per team) motivated students to prepare for the class and contribute to discussions.

*“I felt more motivated because they’re smaller groups. With a bigger group, you kind of hide under the radar and you don’t really need to do as much preparation. TBL motivates you for a lot more preparation to be honest than PBL”.*

The small group size increased dialogue and discussion between team members. A sense of joint purpose was fostered, helping to create a relaxed, collegial atmosphere [19].

*“I actually think it’s nice that the groups were smaller, with four or five, we were actually able to get dialogue going… not interrupted. With the normal PBLs being bigger, sometimes it’s difficult to get a continuous flow. But with this one we could just talk more. I found communication was definitely good, there wasn’t a problem with TBL”.*

### Critical reflection

During PBL, students appreciated the opportunity for the group to work through cases on their own, without direct supervision. Doing so may promote critical reflection, where students are able to reason from their own perspective, and reason with others in order to achieve agreement or confirmation among team members [20].

*“There is something to be said about students being unsupervised in the environment where they think through things on their own …. especially in the first year when – some of us has no background in science at all and maybe our analytical minds aren’t as good as some..”*

In TBL, students felt the individual test and team test at the beginning of class reinforced key concepts for the topic and enabled critical reflection. Learning is supported when there are opportunities to explore and view knowledge in different ways [21].

*“You can test your knowledge of reading and studying because we have the test….I liked the questions. It reinforces the concepts for us. And that enables us to go through and think, how does this work, which is good. I think it would be even better if we had more time to do those 10 questions…more time to discuss the questions”.*

In TBL, an emphasis on active learner involvement, with students tackling problems together, the students’ learning and reflection process was enhanced [1].

*“To see the thought process of other people is really useful. You get to see how other people eliminate answers. How other people work through multiple choice questions.”*

## Discussion

The lens of constructivist learning theory [12] provided a useful framework to understand student perspectives of classroom learning. Four major themes emerged: guided learning, problem solving, collaborative learning, and critical reflection. Students were attracted to the active and collaborative approach of TBL, perceiving the key advantages to be the small group size, the Readiness Assurance Testing process, facilitation by a clinician, an emphasis on basic science concepts, and immediate feedback. The TBL format proved powerful in fostering engagement and learning not always evident within other forms of small group work, such as PBL [22]. However, students expressed a desire for increased opportunity for clinical reasoning within TBL.

### Guided learning

According to constructivist theory, the focus of the learning should be on the student or learner rather than the teacher. The teacher’s role is that of an expert facilitator, to guide the students in taking an active learning role [23]. By following the steps in TBL, one facilitator was readily able to manage four small groups of students simultaneously. Literature suggests that one content-expert facilitator can manage a class of 100 students placed in small groups [24]. It is should be noted that student views on individual tutor performance may not reflect the effectiveness of any individual teaching approach. However, our students expressed preference for the provision of structured guidance, which is limited in PBL. Students noted that an advantage of TBL was the immediate feedback and guidance from the facilitator, despite having double the number of students in the class. Additionally, the Readiness Assurance Process provided the *facilitator* with a means to immediately assess *students’ knowledge* [24] and understanding, and address their specific needs. The importance of providing clinical relevance to medical teaching is frequently highlighted in medical education literature [19, 25]. Students felt that with an experienced senior clinician as a TBL facilitator, the feedback was accurate and clinically relevant.

### Problem solving

Problem solving activities play a key role in engaging students. They need to be of work related relevance, and challenge prior concepts. Guidance from teachers and team members provides scaffolding for learners to build on prior learning [26]. The primary learning objective in TBL methodology is to focus on ensuring students have the opportunity to practice using the core concepts to solve problems [22]. However, in our iteration of TBL, this was deficient. An emphasis on physiology, the readiness assurance test and feedback, as well as time limitations, meant there were insufficient clinical reasoning opportunities within clinical problem solving activities. Our results indicate that while TBL offered advantages in terms of teaching physiology, opportunities were lacking for development of clinical reasoning skills. As noted by Parmelee (2010), significant effort is required to make the problem based activities useful, with an optimal degree of difficulty [27].

### Collaborative learning

Students found that while the PBL format encouraged collaborative learning in groups, it was not uncommon for this learning to *“go off on a massive tangent.”* Students commented on feeling more actively engaged during TBL. This was due to two key elements, including the smaller group size, and the team readiness assurance tests. Completion of TBL tasks, such as tests and problem-solving, required productive team interaction [22]. Additionally, in TBL, students reported feeling motivated to carry out individual preparation for the readiness assurance tests. In the TBL setting, there is little opportunity for individuals to avoid prior preparation and engagement in group activities [27, 28]. Students noted that they were more likely to come to class prepared in TBL, hence the quality of team and class discussions improved. However, in our study we did not have evidence for the stability over a long enough period for the team-development process to come to fruition [27, 29].

### Critical reflection

Opportunities for critical reflection are needed to allow students to make judgements on required modification to their existing knowledge [30]. In TBL, the sequence of the Readiness Assurance Process ensured that students had several opportunities to engage with the content and gauge their own understanding [31]. The tests encouraged self-reflection on knowledge, and also self-reflection on students’ own interactions between group members. Reflection occurred when students compared their understanding to that of their team members during the Team Readiness Assurance process. Students reflected on their own understanding when inconsistencies were exposed. Through discussion to agree on an answer, students were able *“to see the thought process of other people”*, and build one their own understanding. Although reflective practices also occurred through student interaction during PBL sessions, the formal testing procedures in TBLs promoted reflection.

### Limitations

In this qualitative study, we were able to explore in depth the rich experience of 14 students from a theoretical perspective. The six students who did not participate in the focus group may have had different opinions that were not captured by their peer participants. By timing the focus groups immediately following the TBL, we may have influenced students’ perceptions. If we had timed the focus groups at a later date, their views may have altered.

We acknowledge the design and format of both PBL and TBL in this study have been customised to run in a single institution. However, we believe our findings might be helpful to other medical schools investigating the introduction of TBL through pilot studies. We note that we did not provide a control group for the study, which may have provided greater depth to our data and findings.

We acknowledge that the facilitator was a senior medical practitioner and academic, with extensive teaching experience. This may have made the TBL experience more positive for our students (whose focus is on medicine) than might be the case if they were taught by a more junior basic science staff member.

### Next steps

Our study findings indicate that wider scale implementation of TBL, with further modifications is needed before a decision can be made on final changes to teaching methods. Further modifications would include: extended duration of TBL to allow more time for clinical reasoning; and prompts to facilitate clinical reasoning during the TBLs.

## Conclusion

Both TBL and PBL offer effective teaching methods that are based on constructivist learning theory. Our students found their learning experience in TBL to be qualitatively different to their previous experience in PBL, identifying advantages and disadvantages to both. Although the use of TBL required an instructional approach, needing direction from the tutor, it remained student-centred, generating a range of positive outcomes. In particular, TBL resulted in better preparation, immediate feedback on progress, and smaller group size. The findings from our study suggest a hybrid approach that utilises the strengths of both TBL and PBL strategies needs to be considered for wide scale implementation.

## Abbreviations

IRAT: Individual Readiness Assurance Test
PBL: Problem based learning
TBL: Team-based learning
TRAT: Team Readiness Assurance Test
